# Epidemiology and molecular characterization of *Theileria annulata* in cattle from central Khyber Pakhtunkhwa, Pakistan

**DOI:** 10.1371/journal.pone.0249417

**Published:** 2021-09-16

**Authors:** Raqeeb Ullah, Sumaira Shams, Munsif Ali Khan, Sultan Ayaz, Noor ul Akbar, Qeyam ud Din, Adil Khan, Renato Leon, Jehan Zeb

**Affiliations:** 1 Department of Zoology, Abdul Wali Khan University Mardan, Mardan, Pakistan; 2 College of Veterinary Sciences & Animal Husbandry, Abdul Wali Khan University Mardan, Mardan, Pakistan; 3 Department of Zoology, Kohat University of Science and Technology, Kohat, Pakistan; 4 Department of Geography, University of Peshawar, Peshawar, Pakistan; 5 Medical Entomology & Tropical Medicine Laboratory LEMMT, Universidad San Francisco de Quito, Quito, Ecuador; Beni Suef University Faculty of Veterinary Medicine, EGYPT

## Abstract

*Theileria annulata* is a tick-borne hemoprotozoan parasite responsible for tropical theileriosis in the bovine population, which causes substantial economic losses to the livestock sector. The present study has investigated, characterized, and shaped epidemiologic and phylogenetic profiles of *T*. *annulata* infection in the cattle population of central Khyber Pakhtunkhwa, Pakistan. A total of 600 blood samples were collected from cattle. Microscopy and PCR (*18S rRNA* taxonomic marker) assays were performed to detect *T*. *annulata* infection in cattle from the study area. The overall relative prevalence rates of *T*. *annulata* in the examined cattle population were 12.8% (microscopy) and 23.7% (PCR). District-wise analysis (microscopy/PCR) showed that cattle from district Mardan were found more infected (16.0%/28.0%), as compared to cattle from district Charsadda (13.5%/25.5%) and district Peshawar (9.0%/17.5%). Based on host demographic and ecological parameters analysis, theileriosis was found to be higher in young, female, crossbred, freely grazing, tick-infested, and irregular/no acaricides treated cattle. The univariate logistic analysis showed that host age, tick infestation, acaricides use, and feeding method were significant risk factors (P<0.05) whereas multivariate analysis indicated that host age, gender, tick infestation, acaricidal application, and feeding method were potential risk factors (P<0.05) for tropical theileriosis in the cattle population. Phylogenetic and sequence analysis showed that *T*. *annulata 18S rRNA* isolates shared homology and phylogeny with other isolates from Asia and Europe. This study has addressed the epidemiology and phylogeny of *T*. *annulata* circulating in bovid in the study area where gaps were still present. These findings will serve as a baseline and will facilitate future large-scale epidemiological investigations on tropical theileriosis in the cattle population at a national level.

## Introduction

Bovine tropical theileriosis (TT) is a tick-borne disease (TBDs) caused by the hemoprotozoan parasite *Theileria annulate*, which circulates in the bovine population, and results in substantial economic losses to the dairy and livestock industry. It is the cause of high mortality rates and imposes constraints upon the breeding development program, thus significantly reducing its production output [1, 2]. Tropical theileriosis is prevalent over a broad geographic region globally ranging from Asia, the Middle East, and southern Europe, to northern Africa [3, 4]. This pathogen is transmitted by several hard ticks (Ixodidae) species of the genus *Hyalomma* viz. *H*. *anatolicum*, *H*. *lusitanicum*, *H*. *scupense*, and *H*. *dromedarii* [5, 6]. Several studies have confirmed either the occurrence of the mentioned vector species or the presence of *T*. *annulata* in ticks from disease-endemic regions of Pakistan [7–9].

*Theilleria annulata* has been found mainly infecting domestic cattle (*Bos taurus indicus*, *Bos taurus taurus*) and the Asian buffalo (*Bubalus bubalis*) [3, 10]. During feeding on host blood, ticks inoculate *T*. *annulata* sporozoites into the bovid blood where the parasites undergo a complex life cycle; the sporozoites’ invasion of the host mononuclear Leukocytes. Sporozoites subsequently transform into macroschizonts (which replicate in leukocytes) and undergo merogony to develop into merozoites that enter the bloodstream. The merozoites infect the host RBCs and develop into piroplasms. Finally, the infective piroplasms enter the tick during the next blood feeding. Clinical signs associated with TT in bovine are; pyrexia (40–41.5°C), ocular and nasal discharge, enlargement of superficial lymph nodes, dyspnea, leucopenia, anemia, and jaundice [2, 11].

Acute theileriosis in cattle is clinically diagnosed by microscopy which involves either piroplasms detection through the examination of Giemsa-stained peripheral blood smears or macroschizonts in the lymph node biopsy smears [3, 12]. This diagnostic procedure is not enough sensitive to allow the reliable estimation of TT in carrier animals [13]. Additional, diagnostic tools i.e. serodiagnostic assays (IFA and ELISA) and PCR are more sensitive, and specific molecular alternatives for the detection of *T*. *annulata* in the infected animal hosts. The commonly used genetic markers for *T*. *annulata* identification and characterization are; the *18S rRNA* gene, the *T*. *annulata merozoites surface antigen (Tams1*) encoding gene, the *β-tubulin* gene, and the *heat shock protein 70* encoding gene (*HSP70*) [14–19].

Tropical theileriosis is endemic to Pakistan, its etiologic agent is circulating in the bovine population and is principally transmitted by *H*. *anatolicum* tick to them [2, 20]. Plenty of scientific literature has reported TT from certain parts of the country [21]. Based on the microscopic investigation, the mean prevalence rate of TT in the cattle population from Pakistan is 13±5.7%, range 5–24% [9, 22–25]. However molecular diagnostic technique (PCR) has reported a comparatively higher prevalence rate of TT in cattle (38.7±9.9%, range 19–66.1%) [2, 21, 22, 25, 26]. The high prevalence and endemicity of bovine theileriosis to Pakistan was further supported by the availability of its potential vectors i.e. Hyalomma species preferably in arid and semi-arid agro-ecological zones, transmitting *T*. *annulata* in the bovine population [2, 7, 8, 27]. Seasonal patterns have shown that *T*. *annulata* is circulating in the cattle population throughout the year. According to documented proof, the highest prevalence of TT was reported in bovine hosts during the summer season (with peak TT cases in June and July) followed by winter, spring, and autumn seasons respectively [23, 28, 29]. The potential risk factors predicted to be associated with TT in Pakistan and likely facilitating *T*. *annulata* transmission dynamics are cattle age, gender, breed, acaricide application, tick infestation, and farm management system [2, 20, 21].

Despite the availability of epidemiological and phylogenetic data from other agro-climatic regions of Pakistan, this livestock disease has been poorly studied in the north-western part of the country, where a considerable gap remains regarding *T*. *annulata* detection and its epidemiology in the cattle population. The lack of data concerning TT prevalence from the study area results in poor control measures. The study presented herein, detected (microscopy and PCR based) *T*. *annulata* in the cattle population, established its molecular phylogeny based on the study area (central Khyber Pakhtunkhwa, an important hub of cattle farming), and shaped its epidemiologic profile in the different cattle breeds.

## Materials and methods

### Ethics statement

The present study followed the Animal Use Manual of the Pakistan Veterinary Association (PVA) and obtained formal approval from the ethical committee on animal care and use from the Department of Zoology, Abdul Wali Khan University Mardan. All blood samples were collected following standard collection methods and thus avoided stress/harm procedures to the sampled bovine animals.

### Study location

Pakistan is predominantly an agricultural country and divided into five agro-ecological zones based on remote sensing climate compound index-based climatic/aridity data analysis (Drought/hyper-arid, Arid, Humid, Wetland, and Cold drought) [30, 31]. The sampling site (Charsadda 34.1495°N, 71.7428°E; Mardan 34.2062°N, 72.0298°E; and Peshawar 34.0000°N, 71.7500°E located centrally in the Khyber Pakhtunkhwa Province of Pakistan) has a transitional climatic profile i.e. a patchy pattern of semi-arid and humid agro-ecological zones with variable climatic features in certain parts of the study area due to extensive irrigation system [30] and hence, in turn, affect the distribution of tick and TBDs [7].

The study area experiences hot, sometimes very hot, long summer and relatively short cool winter seasons. Summers start from mid-April and peaking in May and early June. Moreover, the summer season is dry and covered with dust storms. There is also an onset of the seasonal effects of monsoons and feature heavy rains on an almost daily basis with a fall in the average daily temperature and a rise in the relative humidity. Whereas the winter seasons are usually short, dry, and tend to be foggy with winter rainfall in January and February. The annual average temperature prevails in the study area is mini-max: 5.0±2.5°C-40.2±5.8°C and mean relative humidity is mini-maxi: 17.7±2.5–65±3.6 [32].

Agriculture is central to the Pakistan economy and contributes a significant part to the country’s GDP (FY 2020, 19.3%). The livestock sector is an essential component of the agricultural industry where over 8 million rural families are actively involved with livestock rearing, production, and development. This activity represents approximately 35–40% of their income source. The livestock sector has contributed 60.6% to the agriculture economy and 11.7% to the country’s GDP during FY 2020 [33]. Cattle population is vital to the livestock sector in Khyber Pakhtunkhwa where 5.97 million animals are being raised; 0.72 million coming from the study area (district Charsadda: 0.24 million, district Mardan: 0.25 million, district Peshawar: 0.23 million) [34].

### Study design, period, and blood sampling strategy

A cross-sectional study was designed to investigate TT in cattle from the study area. The sample size was computed for the cattle population by considering 50% assumed prevalence with a 95% confidence interval and 5% desired precision. The number of collected samples was adjusted for a finite population and correlated with 600 blood samples of cattle. A simple random sampling strategy was adopted for sample collection to allow reliable estimation of TT prevalence in the cattle population from the study area [35].

A total of 30 union councils (included villages and towns with at least one livestock farm) were chosen with a total of 54 farms (18 cattle farms from each district). Six hundred blood samples (2 ml of blood obtained from the jugular vein, n = 200 from each district) were collected from the cattle during 2018 and 2019 from April to September of each year with signed informed consent from the herds’ owners. Necessary information, regarding bovine host age, gender, breed, tick infestation, acaricides practice, and feeding method, was obtained through a predesigned questionnaire from the cattle owners. Collected blood samples were stored in properly labeled blood vacutainers (EDTA tubes, Thermoscientific^™^, USA) at -20°C until further analysis.

### Blood smear microscopic examination

All the collected blood samples were microscopically screened for intra-erythrocytic piroplasms of *T*. *annulata*. A thin blood smear of each blood sample was prepared, air-dried, and fixed in 96% methanol for 5 minutes followed by staining with Giemsa stain (5%) for 30 minutes [36]. Each slide was observed under a 100x objective lens in a compound microscope. More than 30 microscopic fields were examined by a single observer in each smear separately and a slide was considered positive even with the presence of one piroplasm organism. Microscopy-based positive and negative blood samples from each district of the study area were labeled and stored separately for DNA extraction and PCR-based screening. The Cohen’s kappa statistic of reliability was computed to determine the degree of agreement between the blood smear microscopy and PCR. The sensitivity and specificity of blood microscopy in comparison to PCR (reference standard) for the detection of *T*. *annulata* were determined by using the following formulas [37, 38];

$$Sensitivity=\frac{Number\;of\;true\;positives}{Number\;of\;true\;positives\;+\;Number\;of\;false\;negatives}\times 100$$

$$Specifisity=\frac{Number\;of\;true\;negatives}{Number\;of\;true\;negatives\;+\;Number\;of\;false\;positives}\times 100$$

### DNA isolation and PCR

DNA was extracted from each blood sample separately with the help of a Qiagen blood and tissue DNA extraction kit following the manufacturer’s DNA extraction protocol (Qiagen, Hilden, Germany). The DNA concentration in the samples was quantified by OD260 and its purity by the ratio of OD260/OD280 respectively using NanoDrop^™^ 1000 spectrophotometer (NanoDrop Technologies Inc., Wilmington, USA). The DNA samples were then stored at -80°C for subsequent analysis.

Species-specific primers were designed to amplify the hypervariable region (V4) of *T*. *annulata 18S rRNA* genetic marker (Table 1). *Theileria annulata 18S rRNA gene* sequences (n = 100) were downloaded from NCBI GenBank as a single dataset in FASTA format and subjected to multiple sequence alignment using CLUSTALW software [39]. The primers set was picked from aligned sequences and its thermodynamic parameters were calculated using Vector NTI software (Oligo Analyses tools, Thermo Fisher Scientific, US). PCR reaction was performed with a total volume of 25 μl reaction mixture; composed of 1 μl of each primer (forward and reverse), 2 μl of template DNA (OD260: 180±20 μg/μl, OD260/280: 1.85±0.04), 14 μl of DreamTaq Green PCR Master Mix (2X), and 8 μl of PCR grade water. The thermo-cycling conditions were as follows: initial denaturation at 95°C for 5 minutes, followed by 35 cycles of denaturation at 95°C for 30 seconds, annealing at 57°C for 30 seconds, extension at 72°C for 30 seconds, and a final extension at 72°C for 10 minutes. Negative (PCR reaction mixture without template DNA) and positive (previously isolated *T*. *annulata* DNA from the blood of naturally infected cattle provided by veterinary research institute Peshawar Pakistan) controls were included in each run for validation. PCR products were electrophoresed on a 2% agarose gel stained with 2% ethidium bromide and subjected to gel documentation for visualization.

**Table 1:** Species-specific *18S rRNA* gene primers set designed for *T*. *annulata* detection.

Pathogen	Taxonomic marker	Primer sequence 5ʹ-3ʹ	Amplicon	T_m_
*T*. *annulata*	*18S rRNA*	F: AGCCATGCATGTCTAAGTATAAG	894 bp	57°C
		R: CGGTATTTGATATGGCTGATCTC		

### Purification, sequencing, and analysis of PCR products

The PCR products of the *18S rRNA* gene were excised from the gel and purified with a Qiagen PCR Purification Kit (Qiagen, Hilden, Germany) following the manufacturer’s instructions. Fifteen samples (five from each district) of purified PCR products were sequenced by Macrogen, Inc. (Seoul, South Korea). The nucleotide sequences were analyzed and assembled using MEGA X [40]. *Theileria annulata 18S rRNA* homologous (subject sequences) were searched by using NCBI BLASTn [41]. All the subject sequences with query coverage of 99–100% were downloaded for downstream analysis. Query sequences were trimmed to remove unnecessary nucleotides, followed by redundant sequences removal from the data set. Three partial sequences of the *18S rRNA* gene were submitted to NCBI GenBank under accession numbers MW046053, MW046054, and MW046055.

### Phylogenetic and sequence analysis

Partial nucleotide sequences of the *18S rRNA* genetic marker of *T*. *annulata* were aligned using MEGA X [40]. The phylogenetic tree of *T*. *annulata* was constructed using the Neighbor-joining algorithm and the data set was resampled 1000 times for bootstrap values generation. Evolutionary divergence of the present study *T*. *annulata* isolates and previously published sequences from neighboring countries was estimated based on pairwise base difference and complete deletion of positions containing gaps and missing data. Similarly, Pairwise nucleotide percent identity was also computed for the same data set.

### Risk factors investigation

Host demographic and environmental parameters were recorded during sample collection and subjected to univariate and multivariate logistic regression analyses using R program version 3.5.1 (R Development Core Team) to determine their possible role as potential risk factors in TT incidence in the cattle population. P<0.05 for each statistical analysis was considered significant with 95% CI.

## Results

### Demographic profile of cattle population

A total of 600 cattle were examined on 54 livestock farms in the study area. The median herd size of the cattle farm was 10 individuals. Based on the age, more adults were included than young animals (median age = 4 years) whereas gender-wise, more female than male counterparts were present in the study. The cattle population of the study area was composed of three cattle breeds viz. exotic breed (*Bos taurus taurus*), crossbreed (*Bos taurus taurus × Bos taurus indicus*), and indigenous breed (*Bos taurus indicus*) (Table 2).

**Table 2:** Demographic properties of cattle population from the study area.

Demographic Characteristics	Categories	Districts of Khyber Pakhtunkhwa			Total n (%)
		Charsadda n (%)	Mardan n (%)	Peshawar n (%)	
Age	Young	70(35)	74(37)	69(34.5)	213 (35.5)
	Adult	130 (65)	126(63)	131(65.5)	387 (64.5)
Gender	Male	75(37.5)	67(33.5)	64(32.0)	206 (34.3)
	Female	125(62.5)	133(66.5)	136(68)	394 (65.7)
Breed	Indigenous	66(33.0)	64(32.0)	70(35.0)	200 (33.3)
	Cross Breed	67(33.5)	65(32.5.)	64(32)	196 (32.7)
	Exotic Breed	67(33.5)	71(35.5)	66(33)	204 (34.0)

### Blood microscopy

Out of 600 collected blood samples, only 77 (12.8%) were found positive for *T*. *annulata* piroplasms (Table 3). The *κ*-coefficient indicated a moderate level of agreement between the blood smear microscopy and PCR (Table 3). The sensitivity and specificity of the microscopic technique to detect *T*. *annulata* piroplasms was 69% and 100% in comparison to the PCR assay (Tables 3 and 4).

**Table 3:** Comparative analysis of blood microscopy and PCR assay results for *T*. *annulata* detection.

**Diagnostic technique**	**Blood microscopy**		**κ-value**
	**+**	**-**	0.6
**PCR assay**			
**+**	77	65	
**-**	0	458	

+/-: *T*. *annulata* positive/negative samples, κ-value: Cohen’s kappa statistic of reliability between microscopy and PCR

**Table 4:** Relative prevalence, sensitivity, and specificity of blood microscopy and PCR for *T*. *annulata* detection.

Diagnostic technique	Blood samples examined (n)	Positive samples (n)	Sensitivity %	Specificity %	Prevalence %
Blood microscopy	600	77	69	100	12.8
PCR assay	600	142	100	100	23.7

### PCR and sequencing

Based on PCR assay, a total of 142 DNA samples (23.7%, 142/600) were successfully amplified using the *18S rRNA* gene marker. The resultant amplicon size was ~894 bp. No amplification was observed in negative control samples.

### Phylogenetic and sequence analysis

**Fig 1:**
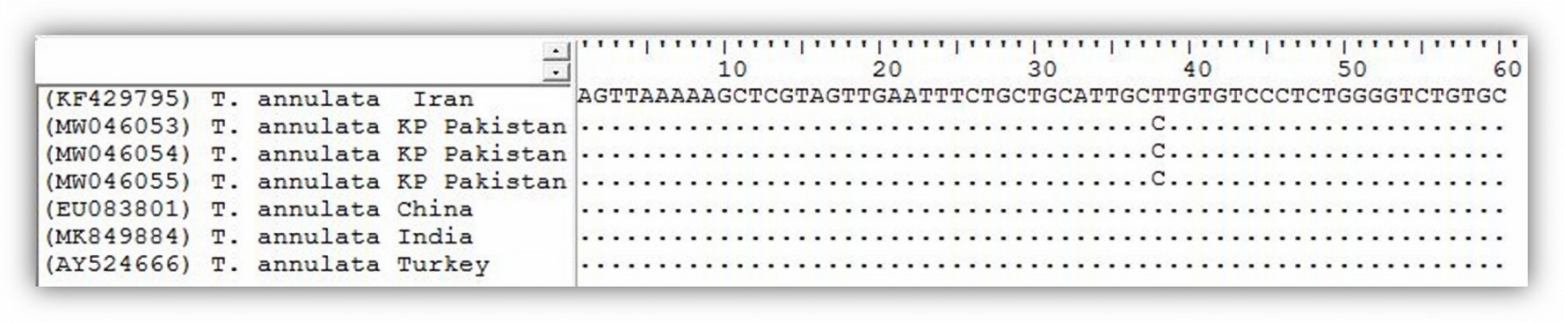
*18S rRNA* gene variable region (V4) in a 60bp nucleotide sequence alignment. Sequence analyses include *T*. *annulata* samples from the present study and published isolates from neighboring countries.

**Fig 2:**
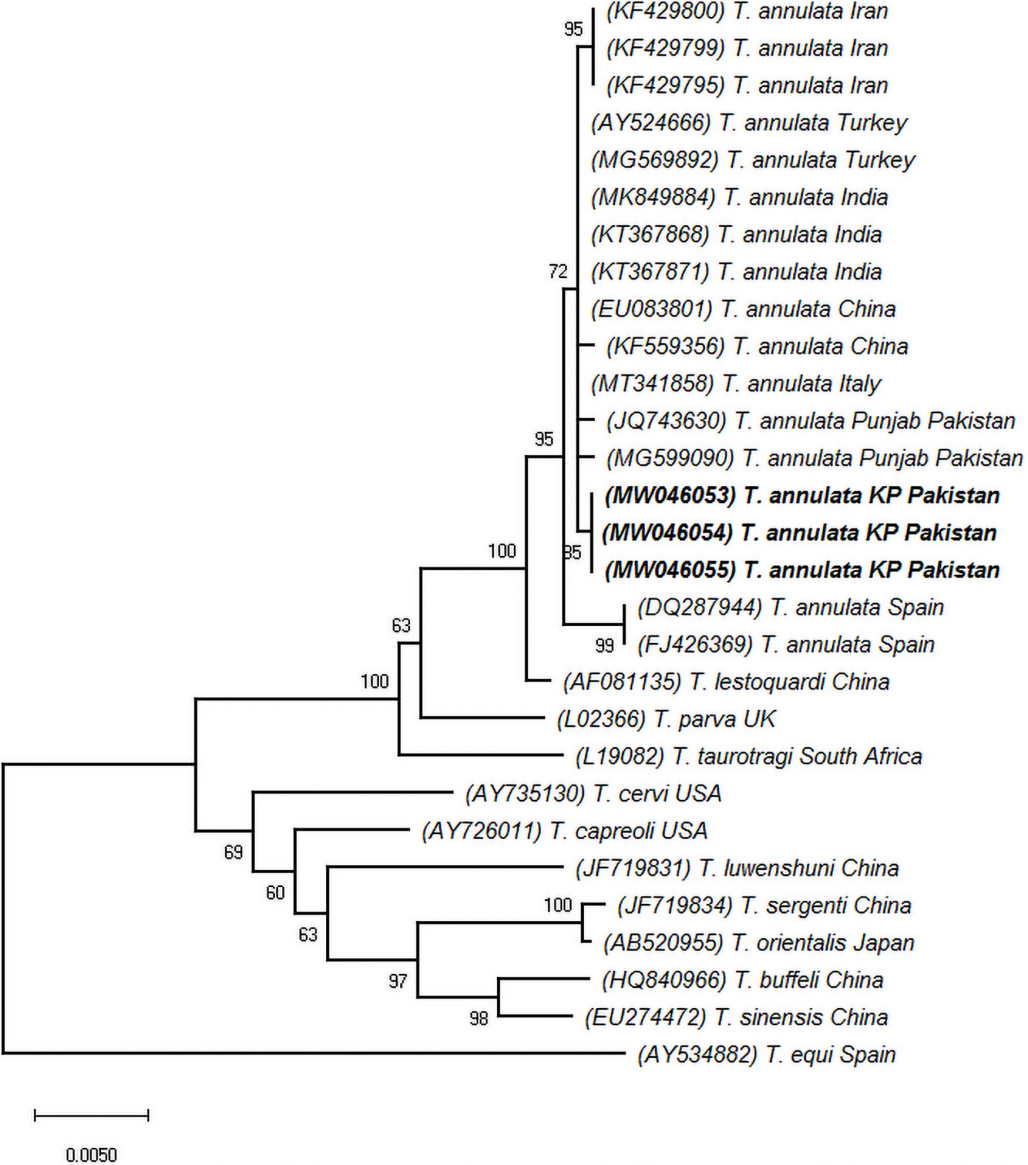
Neighbor-joining algorithm-based phylogenetic tree inferred from *18S rRNA* partial gene sequences of *T*. *annulata* isolates. Parentheses enclosed accession numbers followed by species name and country of origin. *T*. *annulata* isolates from the study area are shown in bold. Scale bar represents nucleotide substitution per site along with bootstrap values at each node. *T*. *equi* (AY534882) was included as an out-group.

The sequence homology search of query sequences showed similarities (Query coverage: 99–100%) with the same isolates reported from Asia, Africa, and Europe; notably from the Punjab province of Pakistan (Accession No. JQ743630, MG599090), India (Accession No. MK849884, KT367871), China (Accession No. EU083801, KF559356), Iran (Accession No. KF429800, KF429799), Turkey (Accession No. AY524666, MG569892) and Spain (Accession No. DQ287944, FJ426369). The phylogenetic tree of *T*. *annulata* isolates was inferred using the *18S rRNA* genetic marker. The neighbor-joining algorithm showed that *T*. *annulata* isolates from the study clustered together with similar isolates from India, Iran, China, Turkey, and Spain (Bootstrap support 72%) as a distinct subclade with 85% bootstrap support which may be due to a single mutation in the V4 region of *18S rRNA* gene (Fig 1). However evolutionary divergence and nucleotide percent identity did not support any new genotype from the study area. Additionally, phylogenetic analysis showed that *T*. *annulata* isolates were the past descendent to the same isolates from India, China, Iran, and Turkey appeared as variants in the study area (Fig 2).

### Heterogeneity analysis of *18S rRNA* gene isolates

Nucleotide sequence heterogeneity analysis showed that *T*. *annulata* isolates were different from each other and to the published isolates by 0–10 bp. Nucleotide percent identity of *18S rRNA* isolates of the present study ranged from 83.6% to 100% (Table 5).

**Table 5:** Evolutionary divergence (lower triangle) and nucleotide percent identity (upper triangle) analysis of *T*. *annulata 18S rRNA* gene isolates (query and subject sequences). Evolutionary divergence analysis estimated the number of pair-wise base pairs differences per site between *18S rRNA* gene sequences. Nucleotide percent identity represented percent identity between *18S rRNA* gene sequences.

***T*. *annulata 18S rRNA* isolates**	**Nucleotide Percent Identity**								
	**Evolutionary Divergence**		**1**	**2**	**3**	**4**	**5**	**6**	
1)MW046053 *T*. *annulata* KP Pakistan		**1**		90.8	100	95.4	95.5	95.5	**1**
2)MW046054 *T*. *annulata* KP Pakistan		**2**	9.0		90.9	89.7	83.6	83.6	**2**
3)MW046055 *T*. *annulata* KP Pakistan		**3**	0.0	9.0		95.4	95.5	95.5	**3**
4)KF429795 *T*. *annulata* Iran		**4**	5.0	9.0	5.0		99.9	99.9	**4**
5)MK849884 *T*. *annulata* India		**5**	4.0	10.0	4.0	1.0		100	**5**
6)EU083801 *T*. *annulata* China		**6**	4.0	10.0	4.0	1.0	0.0		**6**
			**1**	**2**	**3**	**4**	**5**	**6**	

### Prevalence of tropical theileriosis in the cattle population

#### Overall and district wise prevalence

The overall prevalence of TT in the cattle population was 12.8% and 23.7% (blood microscopy and PCR). Blood samples collected during June and July of the study period (2018–19) were mostly found positive for *T*. *annulata* infection suggesting the incidence of TT during these months of the year. Blood microscopic analysis reported the highest prevalence of *T*. *annulata* infection in cattle from district Mardan followed by districts Charsadda and Peshawar. Similarly, PCR-based investigation showed that the TT prevalence rate was comparatively higher in the Mardan district followed by the Charsadda and Peshawar districts (Table 6).

**Table 6:** Overall and district-wise prevalence of *T*. *annulata* infection based on microscopy and PCR assays.

Districts	*T*. *annulata* infection		P-Value
	Microscopy n (%)	PCR n (%)	
Charsadda	27 (13.5)	51 (25.5)	0.01
Mardan	32 (16.0)	56 (28.0)	0.01
Peshawar	18 (9.0)	35 (17.5)	0.01
Total	77 (12.8)	142 (23.7)	0.01

#### Bovine demographic parameters and tropical theileriosis prevalence

*Theileria annulata* prevalence rate varied depending on the host demographic parameters (cattle age, gender, and breed). It was significantly (P<0.05) higher in young cattle (age ≤ 1.5 years) as compared to adult ones (age > 1.5 years). Gender-based analysis showed that females were found more infected than males. Cattle breed was also significantly associated (P<0.05) with TT prevalence. Among the cattle breeds, *T*. *annulata* infection was higher in crossbred cattle (*Bos taurus taurus × Bos taurus indicus*) followed by an exotic (*Bos taurus taurus*: Friesian and Jersey cattle) and indigenous (*Bos taurus indicus*: Sahiwal, Cholistani, and Dhanni cattle) breeds of cattle (Table 7).

**Table 7:** Host demographic parameters based prevalence of *T*. *annulata* infection in cattle population.

Variable	Category	*T*. *annulata* positive % (n)	95% CI	P-Value
Age	Young	40.4 (86)	36.5–43.1	0.01
	Adult	14.5 (56)	10.1–18.6	
Gender	Male	20.9 (43)	15.04–24.2	0.27
	Female	25.1 (99)	21.6–29.6	
Breed	Indigenous	23 (46)	20.5–26.7	0.11
	Crossbreed	25.5 (50)	23.1–28.5	
	Exotic	22.5(46)	20.5–26.7	

#### Environmental factors and tropical theileriosis prevalence

*Theileria annulata* infection differed significantly (P<0.05) in the cattle population from the study area concerning environmental factors (tick infestation, acaricides application, and feeding method). It was more prevalent among cattle with a higher tick infestation as compared to animals having no or a small number of attached ticks. Similarly, cattle treated regularly with acaricides had an infection to a lesser extent as compared to those treated irregularly or not at all. The feeding method showed higher infection of *T*. *annulata* in freely grazing cattle followed by semi grazing and stall feeders respectively (Table 8).

**Table 8:** Environmental factors based prevalence of *T*. *annulata* infection in cattle population.

Variable	Category	*T*. *annulata* positive % (n)	95% CI	P-Value
Acaricide(s) application	No	17.3 (104)	14.2–20.3	0.01
	Irregular	4.7 (28)	3.0–6.4	
	Regular	1.7 (10)	0.7–2.7	
Feeding method	Stall feeding	2.7 (16)	1.3–3.9	0.003
	Semi grazing	4.8 (29)	3.1–6.5	
	Full grazing	16.2 (97)	13.2–19.1	
Tick infestation	Present	21.9 (131)	18.5–25.1	0.001
	Absent	1.8 (11)	0.7–2.9	

### Risk factors for tropical theileriosis in the cattle population

Univariable analysis of demographic and environmental parameters revealed that the statistically significant (P<0.05) risk factors for TT were bovine host age, acaricides use, feeding method, and tick infestation. Cattle breed and gender were found statistically non-significant (P>0.05) (Table 9). Furthermore, the multivariable analysis showed that the host age, gender, acaricides use, feeding method, and tick infestation were potential risk factors for TT in cattle (P<0.05). Cattle breeds were found statistically non-significant (P>0.05) (Table 10).

**Table 9:** Univariate logistic analysis of possible risk factors for tropical theileriosis in cattle.

Category	Variables	*T*. *annulata* positive (%)	OR (95% CI)	P-Value
Age	Young	14.3	4.0 (2.7–5.9)	0.001
	Adult	9.4		
Gender	Male	7.1	0.8 (0.5–1.2)	0.25
	Female	16.6		
Breed	Indigenous	7.7	1.1 (0.9–1.3)	0.56
	Crossbreed	8.3		
	Exotic	7.8		
Acaricide(s) application	No	17.3	3.4 (2.5–4.6)	0.001
	Irregular	4.7		
	Regular	1.7		
Feeding method	Stall feeding	2.7	0.4 (0.3–0.5)	0.001
	Semi grazing	4.8		
	Full grazing	16.2		
Tick infestation	Present	21.9	37.2 (20.3–75.4)	0.001
	Absent	1.8		

OD: odds ratio, CI: confidence interval at 95%

**Table 10:** Multivariate logistic analysis of possible risk factors for tropical theileriosis in cattle.

Category	Variable	OR (CI 95%)	P-Value
Age	Young	3.9 (2.3–6.6)	0.001
	Adult		
Gender	Male	0.5 (0.3–0.8)	0.006
	Female		
Breed	Indigenous	0.8 (0.6–1.2)	0.31
	Cross		
	Exotic		
Acaricide(s) application	No	1.4 (0.9–2.0)	0.001
	Irregular		
	Regular		
Feeding method	Stall feeding	0.7 (0.5–0.9)	0.01
	Semi grazing		
	Full grazing		
Tick Infestation	Present	25.4 (12.5–56.4)	0.001
	Absent		

OD: odds ratio, CI: confidence interval at 95%

## Discussion

Cattle farming is a key activity and an important part of the livestock sector which plays a crucial role in the agricultural economy of Pakistan. Tick-borne pathogens including *T*. *annulata* are endemic to tropical and subtropical regions of the world including Pakistan and constitute a potential threat to livestock farming (cattle farming). Tropical theileriosis results in considerable economic losses to the livestock industry in developing countries [2, 42]. This disease is important in Pakistan and it was estimated that 49.6 million cattle are at risk of developing *T*. *annulata* infection [2, 43].

This study has identified and reported *T*. *annulata* infection in the cattle population from the study area based on blood smear microscopy and PCR. Giemsa stained blood smear microscopic technique revealed an overall prevalence rate of 12.8% for TT. Our findings are in agreement with Giemsa stained blood smear microscopy-based studies of TT from other agro-ecological zones of the country (TT prevalence: 13%, range 5%-24%) and also with India, Iran, and Sudan (TT prevalence: 8%, range 5–33%) [9, 22–25, 38, 44–48].

Despite the diagnostic artifacts (low sensitivity and specificity: unable to detect Theileria species in carrier state and low parasitemia), the examination of piroplasms in Giemsa-stained blood smears is the most frequently used diagnostic technique in veterinary clinical settings to identify acute TT in disease-endemic regions of Pakistan. This is due to the unavailability of molecular and serological diagnostics and resource limitations in most rural veterinary hospitals and clinical settings [49]. The relative sensitivity and specificity of the blood microscopy, as a diagnostic technique, validated previous findings [38, 45]. However, the presence of artifacts, the destruction of the piroplasmic form due to hemolysis (RBCs), thick smear formation, improper staining, lack of microscopic expertise, and low parasitemia, are all the factors that diminish its sensitivity [37, 50].

The description of the microscopical detection of Theileria species solely on the basis of morphology of the piroplasm and macroschizont stages in Giemsa stained blood smears is a challenging task and should include the differential diagnosis criteria for other morphologically related parasites if mixed infections are present [51]. Therefore more sensitive and reliable diagnostic alternative (PCR) was also performed for *T*. *annulata* detection in bovine hosts [12, 25].

The PCR-based report showed that TT was 23.7% prevalent in the cattle population from the study areas. The presence of *T*. *annulata* in cattle was also reported from other geographic regions of the country by using PCR and agreed with the expected value range (19 to 66.1%) of TT prevalence for endemic regions [2, 21, 22, 25, 26]. These findings are also comparable with the reports about *T*. *annulata* detection through PCR from India (Punjab and Odisha) and Egypt that reported 29.26%, 54.86%, and 10.25% TT prevalence rate in cattle respectively [47, 48, 52]. Variability in TT prevalence rate from diverse geographic localities of the world might be attributed to the differences in animal demographic (age, gender) and ecological parameters (agro-ecological zone, irregular application of acaricides and antiprotozoal drugs, management practices, etc.) [52]. It has been reported that singleplex PCR targeting the *18S rRNA* genetic markers possess good analytical sensitivity and specificity as compared to other taxonomic markers to identify multiple piroplasms species [11] and that’s why the present study has preferred the *18S rRNA* genetic marker for *T*. *annulata* detection in cattle population.

Both diagnostic techniques (microscopy and PCR) were compared for TT during the present study. The relative prevalence of TT was 12.8% (microscopy) and 23.7% (PCR). PCR was found more sensitive than microscopy for the detection of hemoprotozoan parasites in cattle. However, the κ-coefficient showed a moderate level of agreement between the microscopy and PCR. A similar comparison was made by multiple surveys from other agro-ecological zones of Pakistan [23, 25, 53]. According to them, PCR was found a more sensitive and accurate diagnostic tool (TT prevalence rate: 19%, 23%, 33.7%) in comparison to microscopy (TT prevalence rate: 3%, 5.2%, 11%) for detection of *T*. *annulata* infection in the cattle population.

District-wise analysis showed that the TT prevalence rate was comparatively higher in cattle population from district Mardan in comparison with districts Charsadda and Peshawar. The differences in TT prevalence among the districts were statistically significant (P<0.05). These variations in TT prevalence rate from the study area may be attributed to the differences in the micro and macro-climatic conditions of the different agro-ecological zones that in turn affect the bionomics of the tick vectors; favoring tick fecundity, distribution, abundance, activity, and ultimately tick-borne pathogens transmission dynamics [2, 7, 23].

Tropical theileriosis also varied in the cattle population with respect to host demographic parameters from the study area (P<0.05). Age-wise analysis showed that young cattle were found more affected by TT (40.4%) as compared to adults (14.5%). Age as risk factor also showed significant results (univariate: P<0.05, OR: 3.9, 95% CI: 2.7–5.9; multivariate: P<0.05, OR: 3.9, 95% CI: 2.3–6.6). These findings are consistent with scientific investigations [2, 20, 29] that reported a similar profile of TT from semi-arid (young: 28%, adult: 27%) and arid (young: 61.54%, adult: 31.91%) agro-climatic regions of the country. The reason for the higher prevalence in young cattle could be the negligence of young ones and taking more care of adult cattle particularly dairy cows by the herds’ owner. Furthermore, the immune system is not fully developed in young cattle to combat *T*. *annulata* infection [54]. On the other hand, a lower prevalence rate of TT in older animals may be due to their multiple recurrent infections and the development of concomitant immunity during their lifetime [6, 17].

During the present study, the gender-based analysis indicated that TT was more prevalent in female cattle (25.1%) as compared to male counterparts (20.9%). The multivariate logistic regression analysis also supported host gender as a potential risk factor for *T*. *annulata* infection (multivariate: P<0.05, OR: 0.5, 95% CI: 0.3–0.8). These results are in line with the findings [2, 22, 25, 48] that reported higher TT prevalence in female cattle (36%, 34.8%) than male (13%, 5.8%), and host gender as a potential risk factor for TT prevalence in the bovine population [20, 28]. The possible explanation for the higher prevalence rate of TT in dams is that they have more hormonal fluctuation, weaken/disturbed immune response during the gestation or lactation period, and carrying more ticks that make them prone to TT incidence [28, 48, 55]. Therefore female cattle need more attention to reduce the risk of being infected with *T*. *annulata* [2, 20, 56].

A higher prevalence of TT was observed in the cross (25.5%) and exotic breeds (23%) of cattle as compared to the indigenous breed (22.5%). These findings are parallel to the previous reports [2, 20, 48] that reported a higher prevalence of TT in the crossbred cattle (14.7%, 39.84%) followed by exotic (13.6%, 28.92%) and indigenous breeds (1.6%, 11.58%) respectively. Cattle breed as potential risk factor for TT prevalence was found non-significant (univariate: P>0.05, OR: 1.1, 95% CI: 0.9–1.3; multivariate: P>0.05, OR: 0.8, 95% CI: 0.6–1.2). The low prevalence rates of TT in indigenous cattle as compared to cross and exotic breeds are characterized by lower acute-phase protein responses controlled by macrophage cytokines, enzootic stability, and a lower chance of tick infestation due to a high level of resistance to ticks as a result of long term exposure to vector ticks over generations [48, 57, 58]. Furthermore, acclimatization of the indigenous breed to the local environment would render them more hardy and resistant to stressors that could predispose them to infection [23, 28].

The prevalence rate of TT also varied in the cattle population concerning environmental factors i.e. acaricides treatment of the cattle, grazing practice, and tick infestation. Tropical theileriosis was significantly (P<0.05) linked with acaricides application on cattle. The infection was higher in cattle with no/irregular acaricides treatment for tick removal as compared to regularly treated cattle. These findings corroborate published studies from other parts of the country and elsewhere [2, 20, 47, 59], that reported lower *T*. *annulata* infection rate in herds where cattle were regularly treated with acaricides for ticks control. Acaricides application was also evaluated as potential risk factor that influence TT prevalence in the cattle population (univariate: P<0.05, OR: 3.4, 95% CI: 2.5–4.6; multivariate: P<0.05, OR: 1.4, 95% CI: 0.9–2.0). Acaricides application as a potential risk factor for TT agreed with the assessment of the published literature from other agro-ecological zones of the country, which presented that regular and proper acaricidal application on cattle could control tick vector and ultimately TT incidence in the endemic region [2, 20, 45].

The grazing practice of cattle was significantly linked (P<0.05) with the prevalence rate of TT in bovine. Tropical theileriosis was least prevalent in cattle with stall feeding activity (kept in farms with forage availability) as compared to cattle with semi-grazing and full grazing practices. These findings are identical with reports about the impact of grazing practice on TT prevalence in bovine animals [2, 20, 47, 56]. Logistic regression analyses also indicated that the feeding method is the critical risk factor influencing TT prevalence (univariate: P<0.05, OR: 0.4 95% CI: 0.3–0.5; multivariate: P<0.05, OR: 0.7, 95% CI: 0.5–0.9). Similar results were also reported from other parts of the country but least support is available to feeding habits as a potential risk factor for TT in the country [2, 8]. It is also known that some farmers from the study area allow their cattle free grazing that increases contact frequency with animals from other herds, facilitating ticks’ exchange that results in increased *T*. *annulata* infection. However stall feeders’ cattle that are kept in fences/farms and are not allowed to roam freely, reduce their exposure to tick sources like other tick-infested animals, plants and grasses in the grazing field where ticks ambush and wait for attachment to a bovine host [2, 60].

During the present study, it was reported that most of the ticks-infested cattle were found infected with *T*. *annulata* as compared to cattle with minimal or no tick infestation (P<0.05). Tick infestation on cattle as a potent risk factor also affected *T*. *annulata* transmission dynamics (univariate: P<0.05, OR: 37.2, 95% CI: 20.3–75.4; multivariate: P<0.05, OR: 25.4, 95% CI: 12.5–56.4). Our results support previously published literature from Pakistan and elsewhere about tick infestation and its possible impact on TT prevalence pattern in cattle as a potential risk factor [2, 20, 27, 43, 56, 61–63]. Published literature indicates that more frequent transmission of *T*. *annulata* occurs when a suitable vector and infected host exist in close proximity [45]. In Pakistan, *T*. *annulata* is principally transmitted by *H*. *anatolicum* to bovine animals. Other studies from Pakistan further validated the presence of *T*. *annulata* in *H*. *anatolicum* (collected from cattle) and bovine host [27, 43].

*Theileria annulata* evolutionary history was inferred through *18S rRNA* taxonomic marker. Homology searches of the *18S rRNA* isolates shared 99–100% similarities with local and global isolates published in NCBI GenBank. The neighbor-joining algorithm grouped the present study *18S rRNA* isolates into *T*. *annulata* clade, which include similar isolates published from other agro-ecological zones of Pakistan, Asia, and Europe; suggesting that these are closely related genetic variants. Similarly, *18S rRNA* genetic marker has been previously used by several studies in Pakistan and elsewhere globally to identify and establish the phylogenetic profile of *T*. *annulata* circulating in bovine hosts [2, 26, 64–66]. Our findings support and validate them. Target amplification of the hypervariable V4 region of the *18S rRNA* gene is a preferred option to accurately identify, classify and explore the population structures of the piroplasm parasites [67, 68].

## Conclusion

This research work investigated the molecular epidemiology of *T*. *annulata* in the cattle population from central Khyber Pakhtunkhwa Pakistan and inferred the evolutionary history of *T*. *annulata* based on an *18S rRNA* taxonomic marker. *Theileria annulata* isolates shared similarities and phylogeny with similar sequences reported from China, India, Iran, Turkey, and Spain. Additionally, the epidemiologic profile of *T*. *annulata* in cattle breeds was assessed and potential risk factors for tropical theileriosis were also determined. This research work has provided data on *T*. *annulata* phylogeny and epidemiology in bovine hosts from central Khyber Pakhtunkhwa Pakistan. These findings will serve as a baseline and will facilitate future large-scale epidemiological investigations on TT at local and global levels.
