# Supplementary material for: Epidemiology and molecular characterization of Theileria annulata in cattle from central Khyber Pakhtunkhwa, Pakistan
Source: PLoS One. 2021 Sep 16;16(9):e0249417. doi: 10.1371/journal.pone.0249417 (PMC8445462; doi:10.1371/journal.pone.0249417)
Supplement: Attachment — Submitted filename: PONE_2021.docx [file pone.0249417.s001.docx]

lines 54-57: the authors state that serological tests for the diagnosis of theielriosis are not very sensitive and not very specific, actually the statement is not correct because according to the OIE manual Chapter 11.10. - OIE of terrestrial animals, the IFA test has good diagnostic reliability.

Line 78: In this section, it is important to give more information about the climate of the three regions studied. In fact, theileriosis can have seasonal recrudescences so it is important that sampling in the three study areas occurred in the same season to support the discussion. For these reasons it would be appropriate to specify the sampling period.

lines 115-124: This entire section could be eliminated by referring only to the instruction manual of the kit used.

lines 127-128: it is required to specify what has been the method used to design the primers (how many sequences have been taken into account, what software has been used etc. ...)

lines 129 - 130. since the extracted DNA was analyzed with the nanodrop it would be appropriate to indicate the concentration of the template and not the microlites, unless the authors started from DNA at the same concentration for all samples.

lines 133- 134. Please give more information about the PCR controls used.

line 171: According to which manual was the presence of T. annulata assessed on optical myroscopy?

lines 205-226: This section adds more information on the control strategies of theileriosis, in fact it is known that to date the vector control is the best strategy for the control of this disease. Interesting is instead the data regarding the feeding of animals.

lines 248 -251: The authors state that the prevalence data obtained by PCR are superimposable to those obtained by the same method elsewhere. It would be appropriate to specify how sensitivity and specificity of their method were assessed.

lines 257 -259: It would be good to reference this statement with recent phylogenetic studies.
