# Supplementary material for: Epidemiology and molecular characterization of Theileria annulata in cattle from central Khyber Pakhtunkhwa, Pakistan
Source: PLoS One. 2021 Sep 16;16(9):e0249417. doi: 10.1371/journal.pone.0249417 (PMC8445462; doi:10.1371/journal.pone.0249417)
Supplement: Attachment — Submitted filename: Response Letter.doc [file pone.0249417.s002.doc]

Dear Editor-in-Chief (Shawky M. Aboelhadid)

Thank you for your letter and the opportunity to revise our paper on ‘EPIDEMIOLOGY AND MOLECULAR CHARACTERIZATION OF THEILERIA ANNULATA IN CATTLE FROM CENTRAL KHYBER PAKHTUNKHWA, PAKISTAN’ The suggestions offered by the reviewers have been immensely helpful, constructive and we also appreciate your insightful comments on revising the methodology portion of the manuscript.

I have included the reviewer comments immediately after this letter and responded individually to each comment, indicating exactly how we addressed each concern or problem and describing the changes we have made. The revisions have been approved by all authors. The changes are marked in the paper as suggested, and the revised manuscript is attached to this email message (track changed and clean copies).

I want to draw your attention to the study area map entitled **“Map of central Khyber Pakhtunkhwa, Pakistan, showing districts where the cattle blood samples were collected”**. This map was removed from the revised manuscript because we did not receive a timely response about the copyright permission from the ESRI organization after multiple contacts with them since April 20, 2021. Alternatively, we provided a piece of noteworthy information about the study area to compensate for the area map.

All other changes have been made accordingly which are listed below.

Sincerely,

Dr Jehan Zeb

Department of Zoology

Abdul Wali Khan University Mardan, Pakistan

Response to the main Concerns by the editor:

Thank you for your assessment. We have made changes accordingly which might be informative.

1. Please ensure that your manuscript meets PLOS ONE's style requirements, including those for file naming. The PLOS ONE style templates can be found at

<https://journals.plos.org/plosone/s/file?id=wjVg/PLOSOne_formatting_sample_main_body.pdf> and

<https://journals.plos.org/plosone/s/file?id=ba62/PLOSOne_formatting_sample_title_authors_affiliations.pdf>

**Response- The manuscript format and style is changed in accordance with the journal format and style.**

Please amend your manuscript to include your abstract after the title page.
**Response- The manuscript amended according to the suggestion.**

2. Thank you for stating the following in the Acknowledgments Section of your manuscript:

[We are grateful to HEC Pakistan for the funding support.]

We note that you have provided funding information that is not currently declared in your Funding Statement. However, funding information should not appear in the Acknowledgments section or other areas of your manuscript. We will only publish funding information present in the Funding Statement section of the online submission form.

Please remove any funding-related text from the manuscript and let us know how you would like to update your Funding Statement. Currently, your Funding Statement reads as follows:

[The author(s) received no specific funding for this work.]

Please include your amended statements within your cover letter; we will change the online submission form on your behalf.

**Response- This section was modified as per suggestion.**

3. Please amend your manuscript to include your abstract after the title page.

**Response- It has been modified accordingly.**

4. We note that Figure 1 in your submission contain map images which may be copyrighted. All PLOS content is published under the Creative Commons Attribution License (CC BY 4.0), which means that the manuscript, images, and Supporting Information files will be freely available online, and any third party is permitted to access, download, copy, distribute, and use these materials in any way, even commercially, with proper attribution. For these reasons, we cannot publish previously copyrighted maps or satellite images created using proprietary data, such as Google software (Google Maps, Street View, and Earth). For more information, see our copyright guidelines: http://journals.plos.org/plosone/s/licenses-and-copyright.

We require you to either (1) present written permission from the copyright holder to publish these figures specifically under the CC BY 4.0 license, or (2) remove the figures from your submission:

1. You may seek permission from the original copyright holder of Figure 1 to publish the content specifically under the CC BY 4.0 license.

We recommend that you contact the original copyright holder with the Content Permission Form (http://journals.plos.org/plosone/s/file?id=7c09/content-permission-form.pdf) and the following text:

“I request permission for the open-access journal PLOS ONE to publish XXX under the Creative Commons Attribution License (CCAL) CC BY 4.0 (http://creativecommons.org/licenses/by/4.0/). Please be aware that this license allows unrestricted use and distribution, even commercially, by third parties. Please reply and provide explicit written permission to publish XXX under a CC BY license and complete the attached form.”

Please upload the completed Content Permission Form or other proof of granted permissions as an "Other" file with your submission.

In the figure caption of the copyrighted figure, please include the following text: “Reprinted from [ref] under a CC BY license, with permission from [name of publisher], original copyright [original copyright year].

**Response-** I want to draw your attention to the study area map entitled “**Map of central Khyber Pakhtunkhwa, Pakistan, showing districts where the cattle blood samples were collected”.** This map was removed from the revised manuscript because we did not receive a timely response about the copyright permission from the ESRI organization after multiple contacts with them since April 20, 2021. Alternatively, we provided a piece of noteworthy information about the study area to compensate for the area map.

Response to Reviewer #1:

**1. Introduction.** The structure of the introduction should be improved in order to show the expected scientific added value of the present work, the state of the art should better highlighted and in particular for the main epidemiological background data regarding the situation of tropical theileriosis (TT) in Pakistan and in the study region: main vectors of TT in Pakistan and in the study region; seasonality of transmission of TT; endemic states, …. This information is essential to appreciate the relevance of the objectives stated in the introduction. The objectives should be ranked according to their contribution to fill the knowledge gap at both international and local levels.

**Response- The entire introduction has been restructured and incorporated the data accordingly.**

**2.** The content of the introduction should be improved by introduction the following modifications: see below

-Line 66; please avoid to use other terminologies instead of TT, accordingly replace Tropical/Mediterranean theileriosis by TT

Line 77: replace the verb attack by invade; furthermore, the sporozoites do not “attack” or invade the erythrocytes;

-Line 79: merozoites are not representing the T. annulate proliferative stage, the macroschizont is the only stage responsible of uncontrolled clonal multiplication of infected mononuclear leucocytes;

-Line 81: please arrange the order of the symptoms of TT according to their frequency, it is classically admitted that hemoglobinuria is not a frequent symptom in TT.
lines 89-91: the authors state that serological tests for the diagnosis of theielriosis are not very sensitive and not very specific, actually the statement is not correct because according to the OIE manual Chapter 11.10. - OIE of terrestrial animals, the IFA test has good diagnostic reliability.
**Response- All of the above contents have been improved and replacements were made as per suggestions.**

**3.** **Materials and Methods.** The sampling strategy should be justified in relation to the main objective stated in the introduction, which is to have more information on TT prevalence.

-The most reliable appreciation of the prevalence is generally depending on the use of representative samples chosen randomly according to census data on cattle population. The authors should show how their sampling strategy is allowing a reliable estimation of TT prevalence which could be extrapolated to the whole cattle population of the study region.

-The study region has a contrasted seasonal climate which influences tick activity and transmission of TT, accordingly the authors should mention the period of their transversal survey. -The recruited sample of 54 farms is totaling 810 bovine heads, among.

Line 118: In this section, it is important to give more information about the climate of the three regions studied. In fact, theileriosis can have seasonal recrudescences so it is important that sampling in the three study areas occurred in the same season to support the discussion. For these reasons it would be appropriate to specify the sampling period.

**Response- All the comments in this section addressed appropriately and the required information provided.**

**4.** Materials and Methods - The description of the microscopical detection of T. annulata on Giemsa stained blood smears should include the differential diagnosis criteria for other morphologically related parasites such as small forms Babesia species, Anaplasma , and other non-malignant Theileria species if present in the study region. -line 161: According to which manual was the presence of T. annulata assessed on optical myroscopy?.
**Response- All the details provided accordingly.**

**5.** Materials and Methods - lines 175-186: This entire section could be eliminated by referring only to the instruction manual of the kit used.

**Response- This section has been removed and referred accordingly.**

**6.** lines 186 - 188. since the extracted DNA was analyzed with the nanodrop it would be appropriate to indicate the concentration of the template and not the microlites, unless the authors started from DNA at the same concentration for all samples.

**Response- This section has been revised and corrected.**

**7.** Lines 190-198: it is required to specify what has been the method used to design the primers (how many sequences have been taken into account, what software has been used etc.)

**Response- This statement has been justified.**

**8.** – Results. The results are globally well presented; however, Table 2 seems to include mis-calculated data that should be corrected; indeed, the percentages of young and adult cattle and of males and females should be calculated within each district, the corresponding percentages shown in the table are referring to the distribution of each sex and age categories between and not within the three sites.

**Response- Table 2 was corrected and miscalculated data was removed.**

**9.** Results. The authors should define clearly what they mean by young animals, they should also give more information on what they call exotic and local cattle breeds.

**Response- It has been explained as requested.**

**10.** Discussion. The discussion is not enough developed, in it is present form it is not valorising the survey results.

-The prevalence is not discussed in terms of significance for TT endemicity states.

**Response- Discussion reframed and discuss in details including prevalence in terms of significance for TT endemicity states and with reference to current status of TT in Pakistan.**

**11-** The results of the risk analysis are not discussed in regard to the bioclimate of the region, the biology of the vector ticks and the transmission patterns of TT in the region or in similar regions in Pakistan or even in neighboring countries with similar contexts. -For instance, the authors have not made any attempt to explain the higher prevalence reported in “young” animals, is that related to the fact that farmers are taking more care for adult cattle and particularly dairy cows?

**Response- Bioclimatic conditions of the region, and the transmission patterns of TT in the region or in similar regions in Pakistan and other countries discussed appropriately.**

**12-** The statistical significance of some risk factors is not discussed.

**Response- All of the concerned part were explained accordingly.**

**13-** Similarly, the discussion has ignored to explain the significant effect of the “feeding method”, is that related to the vector tick biology?

**Response- This portion has been justified accordingly.**

**14-** The results of the phylogenetic analysis are not well discussed.

**Response- Phylogenetic analysis has discussed in detail.**

**15-** In the conclusion, the authors are bringing new developments which should have been included in the discussion, indeed, they are stating that their results could contribute to improve the control of TT. They should show how their results could be applied in practice for this purpose.

**Response- This statement has been justified,**

**16-** The authors state that the prevalence data obtained by PCR are superimposable to those obtained by the same method elsewhere. It would be appropriate to specify how sensitivity and specificity of their method were assessed.

**Response- Specificity and sensitivity are well analysed and specified.**

**17-** It would be good to reference this statement with recent phylogenetic studies.

**Response- It has been made accordingly.**

**18-** The factors retained for risk analysis should be rationally justified on the basis of previous publications and/or by their epidemiological relevance. Furthermore, the authors should give more details on the record of some risk factors and in particular for, i/ ticks presence, is this appreciated in some specific locations preferred

**Response- These factors have been justified accordingly as can be seen in the discussion portion.**

**Response to Reviewer #2:**

The authors present a manuscript that seeks to document the presence of Theileria annulata in a region of Pakistan.

Although the experimental design is correct and supports the rationale of the manuscript, some inaccuracies are present in the text. Regarding the methodology, it is felt that some aspects should be implemented or better specified. Statistical analysis was performed correctly although in some aspects it does not add new knowledge on the study of theileriosis, except for some aspects such as feeding conditions of the animals. The collected data are correctly and clearly presented in the tables. A minimal revision of English is required.

**Response- The methodology portion was corrected and information provided accordingly. Similarly, missing information and English corrections were also made.**
