# Supplementary material for: Epidemiology and molecular characterization of Theileria annulata in cattle from central Khyber Pakhtunkhwa, Pakistan
Source: PLoS One. 2021 Sep 16;16(9):e0249417. doi: 10.1371/journal.pone.0249417 (PMC8445462; doi:10.1371/journal.pone.0249417)
Supplement: Attachment — Submitted filename: Response to Reviewers.doc [file pone.0249417.s003.doc]

R/Dear Academic Editor Dr. Shawky M. Aboelhadid,

Thank you for your letter and the opportunity to revise and submit our manuscript again on ‘EPIDEMIOLOGY AND MOLECULAR CHARACTERIZATION OF THEILERIA ANNULATA IN CATTLE FROM CENTRAL KHYBER PAKHTUNKHWA, PAKISTAN’ The suggestions offered by the reviewer have been immensely helpful and constructive. We are thankful to you, to the reviewers and PLOS ONE scientific platform which support and encourage young researchers’ publications.

We have addressed all the comments, revised and proofread the manuscript together with reference list to exclude any typos if present. Responses to the journal requirements and reviewer’s comments are included immediately after this letter. The revised manuscript is attached to this email message (track changed and clean copies).

Sincerely,

Dr. Jehan Zeb

Department of Zoology

Abdul Wali Khan University Mardan, Pakistan

**Response to the Journal Requirements:**

**1.** Please review your reference list to ensure that it is complete and correct. If you have cited papers that have been retracted, please include the rationale for doing so in the manuscript text, or remove these references and replace them with relevant current references. Any changes to the reference list should be mentioned in the rebuttal letter that accompanies your revised manuscript. If you need to cite a retracted article, indicate the article’s retracted status in the References list and also include a citation and full reference for the retraction notice.

**Response:** The whole reference list is carefully reviewed. No retracted reference was found in the list. The references are arranged according to the standard PLOS ONE style template. The reference section is complete and correct.

**Response to the Reviewer’s Comments**

**Reviewer #1:** The revised manuscript has been significantly improved, the majority of the reviewers’ recommendations were addressed. For this reason, I recommend to accept the present manuscript for publication in PlosOne. In order to optimise the quality of the final version of the manuscript, I will however strongly recommend to the authors, to nclude the following points:

**1.** To mention the tropical theileriosis season(s) in the study region, since this information will position the sampling period (April to September) in regard to the disease season, this information could be relevant to discuss better the sensitivity of blood smears and PCR detection in regard to T. annulata transmission dynamics.

**Response:** The relevant information are addressed in the revised manuscript. The literature about the disease season (s) is inserted in the introduction section and our findings are also explained in the results sections.

**2.** A mention if available in the region or in similar other Pakistani regions on the vector Hyalomma anatolicum ticks dynamics.

**Response:** There is no relevant data available about the vector tick *H. anatolicum* dynamics. All such information will be investigated and addressed soon in our incoming articles.

**3.** To add to the discussion possible reasons explaining the higher prevalence recorded in younger animals.

**Response:** We have provided relevant and possible reasons to justify the higher prevalence recorded in younger animals.

In addition, the authors should take care to make the following corrections:

- **Line 61:** use the valid name of *H. scupense* instead of *H. detritum*.

**Response:** The mentioned species name (*H. detritum*) was replaced with the valid species name *H. scupense*. The synonym is removed.

**- Line 77:** the phrase “This diagnostic procedure has limited use as it cannot detect *T. annulata* in infected carrier animals” is not coherent with the fact that the animals detected in the present study are presumably carriers since the authors did not mention any clinical cases of tropical theileriosis in the surveyed farms. I will recommend to change the phrase as follow: this diagnostic procedure is not enough sensitive to allow a reliable detection of carrier animals.

**Response:** The mentioned sentence is rephrased as suggested by reviewer “this diagnostic procedure is not enough sensitive to allow a reliable detection of TT in carrier animals”.

**- Line 225:** the last phrase is redundant since the same information has been mentioned at lines 222-223.

**Response:** This portion is reshaped by removing the redundant phrase.

**We are thankful to reviewer # 1 for his expert and constructive comments which have improved the quality of our manuscript.**

**Reviewer #2:** The authors followed all indications suggested in the first round of review. All suggestions were properly followed and all requests accommodated.

Response: Thank you sir for providing us the opportunity to revise and improve our manuscript.
