# Supplementary material for: Epidemiology and molecular characterization of Theileria annulata in cattle from central Khyber Pakhtunkhwa, Pakistan
Source: PLoS One. 2021 Sep 16;16(9):e0249417. doi: 10.1371/journal.pone.0249417 (PMC8445462; doi:10.1371/journal.pone.0249417)
Supplement: Peer review history

# Peer review: Decision Letter 0

**DOI:** 10.1371/journal.pone.0249417.r001

**Authors:** Shawky M. Aboelhadid

23 Apr 2021

PONE-D-21-07164

Epidemiology and molecular characterization of Theileria annulata in cattle from central Khyber Pakhtunkhwa, Pakistan

PLOS ONE

Dear Dr.  Jehan Zeb,

Thank you for submitting your manuscript to PLOS ONE. After careful consideration, we feel that it has merit but does not fully meet PLOS ONE’s publication criteria as it currently stands. Therefore, we invite you to submit a revised version of the manuscript that addresses the points raised during the review process.

Please submit your revised manuscript by Jun 07 2021 11:59PM. If you will need more time than this to complete your revisions, please reply to this message or contact the journal office at plosone@plos.org. When you're ready to submit your revision, log on to https://www.editorialmanager.com/pone/ and select the 'Submissions Needing Revision' folder to locate your manuscript file.

Please include the following items when submitting your revised manuscript:

- A rebuttal letter that responds to each point raised by the academic editor and reviewer(s). You should upload this letter as a separate file labeled 'Response to Reviewers'.
- A marked-up copy of your manuscript that highlights changes made to the original version. You should upload this as a separate file labeled 'Revised Manuscript with Track Changes'.
- An unmarked version of your revised paper without tracked changes. You should upload this as a separate file labeled 'Manuscript'.

If you would like to make changes to your financial disclosure, please include your updated statement in your cover letter. Guidelines for resubmitting your figure files are available below the reviewer comments at the end of this letter.

If applicable, we recommend that you deposit your laboratory protocols in protocols.io to enhance the reproducibility of your results. Protocols.io assigns your protocol its own identifier (DOI) so that it can be cited independently in the future. For instructions see: http://journals.plos.org/plosone/s/submission-guidelines#loc-laboratory-protocols. Additionally, PLOS ONE offers an option for publishing peer-reviewed Lab Protocol articles, which describe protocols hosted on protocols.io. Read more information on sharing protocols at https://plos.org/protocols?utm_medium=editorial-email&utm_source=authorletters&utm_campaign=protocols.

We look forward to receiving your revised manuscript.

Kind regards,

Shawky M. Aboelhadid, PhD

Academic Editor

PLOS ONE

Journal Requirements:

When submitting your revision, we need you to address these additional requirements.

1. Please ensure that your manuscript meets PLOS ONE's style requirements, including those for file naming. The PLOS ONE style templates can be found at

https://journals.plos.org/plosone/s/file?id=wjVg/PLOSOne_formatting_sample_main_body.pdf and

https://journals.plos.org/plosone/s/file?id=ba62/PLOSOne_formatting_sample_title_authors_affiliations.pdf

2. Thank you for stating the following in the Acknowledgments Section of your manuscript:

[We are grateful to HEC Pakistan for the funding support.]

We note that you have provided funding information that is not currently declared in your Funding Statement. However, funding information should not appear in the Acknowledgments section or other areas of your manuscript. We will only publish funding information present in the Funding Statement section of the online submission form.

Please remove any funding-related text from the manuscript and let us know how you would like to update your Funding Statement. Currently, your Funding Statement reads as follows:

 [The author(s) received no specific funding for this work.]

Please include your amended statements within your cover letter; we will change the online submission form on your behalf.

3. Please amend your manuscript to include your abstract after the title page.

4. We note that Figure 1 in your submission contain map images which may be copyrighted. All PLOS content is published under the Creative Commons Attribution License (CC BY 4.0), which means that the manuscript, images, and Supporting Information files will be freely available online, and any third party is permitted to access, download, copy, distribute, and use these materials in any way, even commercially, with proper attribution. For these reasons, we cannot publish previously copyrighted maps or satellite images created using proprietary data, such as Google software (Google Maps, Street View, and Earth). For more information, see our copyright guidelines: http://journals.plos.org/plosone/s/licenses-and-copyright.

We require you to either (1) present written permission from the copyright holder to publish these figures specifically under the CC BY 4.0 license, or (2) remove the figures from your submission:

1.    You may seek permission from the original copyright holder of Figure 1 to publish the content specifically under the CC BY 4.0 license. 

We recommend that you contact the original copyright holder with the Content Permission Form (http://journals.plos.org/plosone/s/file?id=7c09/content-permission-form.pdf) and the following text:

“I request permission for the open-access journal PLOS ONE to publish XXX under the Creative Commons Attribution License (CCAL) CC BY 4.0 (http://creativecommons.org/licenses/by/4.0/). Please be aware that this license allows unrestricted use and distribution, even commercially, by third parties. Please reply and provide explicit written permission to publish XXX under a CC BY license and complete the attached form.”

Please upload the completed Content Permission Form or other proof of granted permissions as an "Other" file with your submission.

In the figure caption of the copyrighted figure, please include the following text: “Reprinted from [ref] under a CC BY license, with permission from [name of publisher], original copyright [original copyright year].”

2.    If you are unable to obtain permission from the original copyright holder to publish these figures under the CC BY 4.0 license or if the copyright holder’s requirements are incompatible with the CC BY 4.0 license, please either i) remove the figure or ii) supply a replacement figure that complies with the CC BY 4.0 license. Please check copyright information on all replacement figures and update the figure caption with source information. If applicable, please specify in the figure caption text when a figure is similar but not identical to the original image and is therefore for illustrative purposes only.

The following resources for replacing copyrighted map figures may be helpful:

USGS National Map Viewer (public domain): http://viewer.nationalmap.gov/viewer/

The Gateway to Astronaut Photography of Earth (public domain): http://eol.jsc.nasa.gov/sseop/clickmap/

Maps at the CIA (public domain): https://www.cia.gov/library/publications/the-world-factbook/index.html and https://www.cia.gov/library/publications/cia-maps-publications/index.html

NASA Earth Observatory (public domain): http://earthobservatory.nasa.gov/

Landsat: http://landsat.visibleearth.nasa.gov/

USGS EROS (Earth Resources Observatory and Science (EROS) Center) (public domain): http://eros.usgs.gov/#

Natural Earth (public domain): http://www.naturalearthdata.com/

[Note: HTML markup is below. Please do not edit.]

Reviewers' comments:

Reviewer's Responses to Questions

**Comments to the Author**

1. Is the manuscript technically sound, and do the data support the conclusions?

The manuscript must describe a technically sound piece of scientific research with data that supports the conclusions. Experiments must have been conducted rigorously, with appropriate controls, replication, and sample sizes. The conclusions must be drawn appropriately based on the data presented. 

Reviewer #1: Partly

Reviewer #2: Partly

**********

2. Has the statistical analysis been performed appropriately and rigorously? 

Reviewer #1: Yes

Reviewer #2: Yes

**********

3. Have the authors made all data underlying the findings in their manuscript fully available?

The PLOS Data policy requires authors to make all data underlying the findings described in their manuscript fully available without restriction, with rare exception (please refer to the Data Availability Statement in the manuscript PDF file). The data should be provided as part of the manuscript or its supporting information, or deposited to a public repository. For example, in addition to summary statistics, the data points behind means, medians and variance measures should be available. If there are restrictions on publicly sharing data—e.g. participant privacy or use of data from a third party—those must be specified.

Reviewer #1: Yes

Reviewer #2: Yes

**********

4. Is the manuscript presented in an intelligible fashion and written in standard English?

PLOS ONE does not copyedit accepted manuscripts, so the language in submitted articles must be clear, correct, and unambiguous. Any typographical or grammatical errors should be corrected at revision, so please note any specific errors here.

Reviewer #1: Yes

Reviewer #2: No

**********

5. Review Comments to the Author

Please use the space provided to explain your answers to the questions above. You may also include additional comments for the author, including concerns about dual publication, research ethics, or publication ethics. (Please upload your review as an attachment if it exceeds 20,000 characters)

Reviewer #1: The present manuscript represents an original descriptive survey on tropical theileriosis in the North Western mountainous parts of Pakistan. The manuscript is written in intelligible standard English. The ethics rules are adequately considered. However, as it stands, the present version could not be published in PLOS ONE. In order to be accepted the manuscript should undergo a major revision, its quality could be significantly improved considering the following comments:

INTRODUCTION

The structure of the introduction should be improved in order to show the expected scientific added value of the present work, the state of the art should better highlighted and in particular for the main epidemiological background data regarding the situation of tropical theileriosis (TT) in Pakistan and in the study region: main vectors of TT in Pakistan and in the study region; seasonality of transmission of TT ; endemic states, …. This information is essential to appreciate the relevance of the objectives stated in the introduction. The objectives should be ranked according to their contribution to fill the knowledge gap at both international and local levels.

The content of the introduction should be improved by introduction the following modifications:

-Line 36; please avoid to use other terminologies instead of TT, accordingly replace Tropical/Mediterranean theileriosis by TT;

-Line 45: replace the verb attack by invade; furthermore, the sporozoites do not “attack” or invade the erythrocytes;

-Line 48: merozoites are not representing the T. annulate proliferative stage, the macroschizont is the only stage responsible of uncontrolled clonal multiplication of infected mononuclear leucocytes;

-Line 51: please arrange the order of the symptoms of TT according to their frequency, it is classically admitted that hemoglobinuria is not a frequent symptom in TT.

MATERIAL AND METHODS

The bioclimatic main features of the study region should be described since they modulate vector ticks activity and hence TT transmission dynamics.

The sampling strategy should be justified in relation to the main objective stated in the introduction, which is to have more information on TT prevalence. The most reliable appreciation of the prevalence is generally depending on the use of representative samples chosen randomly according to census data on cattle population. The authors should show how their sampling strategy is allowing a reliable estimation of TT prevalence which could be extrapolated to the whole cattle population of the study region.

The study region has a contrasted seasonal climate which influences tick activity and transmission of TT, accordingly the authors should mention the period of their transversal survey.

The recruited sample of 54 farms is totaling 810 bovine heads, among these only 600 animals were subject to blood sampling, accordingly the authors should mention the exclusion criteria /reasons for the 210 remaining heads.

The authors should define clearly what they mean by young animals, they should also give more information on what they call exotic and local cattle breeds.

The description of the microscopical detection of T. annulata on Giemsa stained blood smears should include the differential diagnosis criteria for other morphologically related parasites such as small forms Babesia species, Anaplasma , and other non-malignant Theileria species if present in the study region.

The factors retained for risk analysis should be rationally justified on the basis of previous publications and/or by their epidemiological relevance. Furthermore, the authors should give more details on the record of some risk factors and in particular for, i/ ticks presence, is this appreciated in some specific locations preferred by the Hyalomma genus or is it a generic question? ii/ regular and irregular tick treatment, technically what how are they defined?

RESULTS

The results are globally well presented; however, Table 2 seems to include mis-calculated data that should be corrected; indeed, the percentages of young and adult cattle and of males and females should be calculated within each district, the corresponding percentages shown in the table are referring to the distribution of each sex and age categories between and not within the three sites.

DISCUSSION

The discussion is not enough developed, in it is present form it is not valorizing the survey results.

The prevalence is not discussed in terms of significance for TT endemicity states.

The results of the risk analysis are not discussed in regard to the bioclimate of the region, the biology of the vector ticks and the transmission patterns of TT in the region or in similar regions in Pakistan or even in neighboring countries with similar contexts. For instance, the authors have not made any attempt to explain the higher prevalence reported in “young” animals, is that related to the fact that farmers are taking more care for adult cattle and particularly dairy cows? The statistical significance of some risk factors is not discussed. Similarly, the discussion has ignored to explain the significant effect of the “feeding method”, is that related to the vector tick biology?

The results of the phylogenetic analysis are not well discussed.

In the conclusion, the authors are bringing new developments which should have been included in the discussion, indeed, they are stating that their results could contribute to improve the control of TT. They should show how their results could be applied in practice for this purpose.

Reviewer #2: The authors present a manuscript that seeks to document the presence of Theileria annulata in a region of Pakistan.

Although the experimental design is correct and supports the rationale of the manuscript, some inaccuracies are present in the text. Regarding the methodology, it is felt that some aspects should be implemented or better specified. Statistical analysis was performed correctly although in some aspects it does not add new knowledge on the study of theileriosis, except for some aspects such as feeding conditions of the animals. The collected data are correctly and clearly presented in the tables. A minimal revision of English is required.

**********

6. PLOS authors have the option to publish the peer review history of their article (what does this mean?). If published, this will include your full peer review and any attached files.

If you choose “no”, your identity will remain anonymous but your review may still be made public.

**Do you want your identity to be public for this peer review?** For information about this choice, including consent withdrawal, please see our Privacy Policy.

Reviewer #1: No

Reviewer #2: No

[NOTE: If reviewer comments were submitted as an attachment file, they will be attached to this email and accessible via the submission site. Please log into your account, locate the manuscript record, and check for the action link "View Attachments". If this link does not appear, there are no attachment files.]

While revising your submission, please upload your figure files to the Preflight Analysis and Conversion Engine (PACE) digital diagnostic tool, https://pacev2.apexcovantage.com/. PACE helps ensure that figures meet PLOS requirements. To use PACE, you must first register as a user. Registration is free. Then, login and navigate to the UPLOAD tab, where you will find detailed instructions on how to use the tool. If you encounter any issues or have any questions when using PACE, please email PLOS at figures@plos.org. Please note that Supporting Information files do not need this step.

Attachment Submitted filename: PONE_2021.docx Click here for additional data file.

# Author comment: Author response to Decision Letter 0

**DOI:** 10.1371/journal.pone.0249417.r002

29 Jun 2021

Dear Editor-in-Chief (Shawky M. Aboelhadid)

Thank you for your letter and the opportunity to revise our paper on ‘EPIDEMIOLOGY AND MOLECULAR CHARACTERIZATION OF THEILERIA ANNULATA IN CATTLE FROM CENTRAL KHYBER PAKHTUNKHWA, PAKISTAN’ The suggestions offered by the reviewers have been immensely helpful, constructive and we also appreciate your insightful comments on revising the methodology portion of the manuscript.

I have included the reviewer comments immediately after this letter and responded individually to each comment, indicating exactly how we addressed each concern or problem and describing the changes we have made. The revisions have been approved by all authors. The changes are marked in the paper as suggested, and the revised manuscript is attached to this email message (track changed and clean copies).

All other changes have been made accordingly which are listed below.

Sincerely,

Dr Jehan Zeb 

Department of Zoology

Abdul Wali Khan University Mardan, Pakistan

Response to the main Concerns by the editor:

Thank you for your assessment. We have made changes accordingly which might be informative.

1. Please ensure that your manuscript meets PLOS ONE's style requirements, including those for file naming. The PLOS ONE style templates can be found at

https://journals.plos.org/plosone/s/file?id=wjVg/PLOSOne_formatting_sample_main_body.pdf and

https://journals.plos.org/plosone/s/file?id=ba62/PLOSOne_formatting_sample_title_authors_affiliations.pdf

Response. The manuscript format and style is changed in accordance with the journal format and style.

Please amend your manuscript to include your abstract after the title page.

Response- The manuscript amended according to the suggestion.

2. Thank you for stating the following in the Acknowledgments Section of your manuscript:

[We are grateful to HEC Pakistan for the funding support.]

We note that you have provided funding information that is not currently declared in your Funding Statement. However, funding information should not appear in the Acknowledgments section or other areas of your manuscript. We will only publish funding information present in the Funding Statement section of the online submission form.

Please remove any funding-related text from the manuscript and let us know how you would like to update your Funding Statement. Currently, your Funding Statement reads as follows:

 [The author(s) received no specific funding for this work.]

Please include your amended statements within your cover letter; we will change the online submission form on your behalf.

Response. This section was modified as per suggestion. 

3. Please amend your manuscript to include your abstract after the title page.

Response. It has been modified accordingly.

4. We note that Figure 1 in your submission contain map images which may be copyrighted. All PLOS content is published under the Creative Commons Attribution License (CC BY 4.0), which means that the manuscript, images, and Supporting Information files will be freely available online, and any third party is permitted to access, download, copy, distribute, and use these materials in any way, even commercially, with proper attribution. For these reasons, we cannot publish previously copyrighted maps or satellite images created using proprietary data, such as Google software (Google Maps, Street View, and Earth). For more information, see our copyright guidelines: http://journals.plos.org/plosone/s/licenses-and-copyright.

We require you to either (1) present written permission from the copyright holder to publish these figures specifically under the CC BY 4.0 license, or (2) remove the figures from your submission:

1. You may seek permission from the original copyright holder of Figure 1 to publish the content specifically under the CC BY 4.0 license. 

We recommend that you contact the original copyright holder with the Content Permission Form (http://journals.plos.org/plosone/s/file?id=7c09/content-permission-form.pdf) and the following text:

“I request permission for the open-access journal PLOS ONE to publish XXX under the Creative Commons Attribution License (CCAL) CC BY 4.0 (http://creativecommons.org/licenses/by/4.0/). Please be aware that this license allows unrestricted use and distribution, even commercially, by third parties. Please reply and provide explicit written permission to publish XXX under a CC BY license and complete the attached form.”

Please upload the completed Content Permission Form or other proof of granted permissions as an "Other" file with your submission.

In the figure caption of the copyrighted figure, please include the following text: “Reprinted from [ref] under a CC BY license, with permission from [name of publisher], original copyright [original copyright year].

Response. The map was designed by one of our co-authors and he agrees that the figure be published in PlosOne. Also we have checked with ESRI and that further permission from ESRI is not required The copyright permission was taken from the original copy holder with the following statement.

“I have no objection to publish the "Figure 1. Map of Central Khyber Pakhtunkhwa, Pakistan and the districts where the cattle blood samples were collected" under the Creative Commons Attribution License”.

Response to the Reviewer’s Comments

Reviewer #1: 

1. Introduction. The structure of the introduction should be improved in order to show the expected scientific added value of the present work, the state of the art should better highlighted and in particular for the main epidemiological background data regarding the situation of tropical theileriosis (TT) in Pakistan and in the study region: main vectors of TT in Pakistan and in the study region; seasonality of transmission of TT; endemic states, …. This information is essential to appreciate the relevance of the objectives stated in the introduction. The objectives should be ranked according to their contribution to fill the knowledge gap at both international and local levels.

Response- The entire introduction has been restructured and incorporated the data accordingly.

2. The content of the introduction should be improved by introduction the following modifications: see below

-Line 66; please avoid to use other terminologies instead of TT, accordingly replace Tropical/Mediterranean theileriosis by TT

Line 77: replace the verb attack by invade; furthermore, the sporozoites do not “attack” or invade the erythrocytes;

-Line 79: merozoites are not representing the T. annulate proliferative stage, the macroschizont is the only stage responsible of uncontrolled clonal multiplication of infected mononuclear leucocytes;

-Line 81: please arrange the order of the symptoms of TT according to their frequency, it is classically admitted that hemoglobinuria is not a frequent symptom in TT.

lines 89-91: the authors state that serological tests for the diagnosis of theielriosis are not very sensitive and not very specific, actually the statement is not correct because according to the OIE manual Chapter 11.10. - OIE of terrestrial animals, the IFA test has good diagnostic reliability.

Response- All of the above contents have been improved and replacements were made as per suggestions.

3. Materials and Methods. The sampling strategy should be justified in relation to the main objective stated in the introduction, which is to have more information on TT prevalence. 

-The most reliable appreciation of the prevalence is generally depending on the use of representative samples chosen randomly according to census data on cattle population. The authors should show how their sampling strategy is allowing a reliable estimation of TT prevalence which could be extrapolated to the whole cattle population of the study region.

-The study region has a contrasted seasonal climate which influences tick activity and transmission of TT, accordingly the authors should mention the period of their transversal survey. -The recruited sample of 54 farms is totaling 810 bovine heads, among.

Line 118: In this section, it is important to give more information about the climate of the three regions studied. In fact, theileriosis can have seasonal recrudescences so it is important that sampling in the three study areas occurred in the same season to support the discussion. For these reasons it would be appropriate to specify the sampling period. 

Response- All the comments in this section addressed appropriately and the required information provided. 

4. Materials and Methods - The description of the microscopical detection of T. annulata on Giemsa stained blood smears should include the differential diagnosis criteria for other morphologically related parasites such as small forms Babesia species, Anaplasma , and other non-malignant Theileria species if present in the study region. -line 161: According to which manual was the presence of T. annulata assessed on optical myroscopy?.

Response- All the details provided accordingly.

5. Materials and Methods - lines 175-186: This entire section could be eliminated by referring only to the instruction manual of the kit used.

Response- This section has been removed and referred accordingly.

6. lines 186 - 188. since the extracted DNA was analyzed with the nanodrop it would be appropriate to indicate the concentration of the template and not the microlites, unless the authors started from DNA at the same concentration for all samples.

Response- This section has been corrected.

7. lines 190-198: it is required to specify what has been the method used to design the primers (how many sequences have been taken into account, what software has been used etc. ...)

Response. This statement has been justified. 

8. -The results are globally well presented; however, Table 2 seems to include mis-calculated data that should be corrected; indeed, the percentages of young and adult cattle and of males and females should be calculated within each district, the corresponding percentages shown in the table are referring to the distribution of each sex and age categories between and not within the three sites.

Response. Table 2 was corrected and miscalculated data was removed.

9. The authors should define clearly what they mean by young animals, they should also give more information on what they call exotic and local cattle breeds.

Response. It has been explained as requested. 

10. The discussion is not enough developed, in it is present form it is not valorizing the survey results.

-The prevalence is not discussed in terms of significance for TT endemicity states. 

Response. Discussion reframed and discuss in details including prevalence in terms of significance for TT endemicity states and with reference to current status of TT in Pakistan.

11- The results of the risk analysis are not discussed in regard to the bioclimate of the region, the biology of the vector ticks and the transmission patterns of TT in the region or in similar regions in Pakistan or even in neighboring countries with similar contexts. -For instance, the authors have not made any attempt to explain the higher prevalence reported in “young” animals, is that related to the fact that farmers are taking more care for adult cattle and particularly dairy cows? 

Response. Bioclimatic conditions of the region, and the transmission patterns of TT in the region or in similar regions in Pakistan and other countries discussed appropriately.

12- The statistical significance of some risk factors is not discussed. 

Response. All of the concerned part were explained accordingly. 

13- Similarly, the discussion has ignored to explain the significant effect of the “feeding method”, is that related to the vector tick biology?

Response. This portion has been justified accordingly. 

14- The results of the phylogenetic analysis are not well discussed.

Response. Phylogenetic analysis has discussed in detail.

15- In the conclusion, the authors are bringing new developments which should have been included in the discussion, indeed, they are stating that their results could contribute to improve the control of TT. They should show how their results could be applied in practice for this purpose.

Response. This statement has been justified, 

16- The authors state that the prevalence data obtained by PCR are superimposable to those obtained by the same method elsewhere. It would be appropriate to specify how sensitivity and specificity of their method were assessed.

Response. Specificity and sensitivity are well analysed and specified. 

17- It would be good to reference this statement with recent phylogenetic studies.

Response. It has been made accordingly. 

18- The factors retained for risk analysis should be rationally justified on the basis of previous publications and/or by their epidemiological relevance. Furthermore, the authors should give more details on the record of some risk factors and in particular for, i/ ticks presence, is this appreciated in some specific locations preferred

Response. These factors have been justified accordingly as can be seen in the discussion portion. 

Reviewer #2: 

The authors present a manuscript that seeks to document the presence of Theileria annulata in a region of Pakistan.

Although the experimental design is correct and supports the rationale of the manuscript, some inaccuracies are present in the text. Regarding the methodology, it is felt that some aspects should be implemented or better specified. Statistical analysis was performed correctly although in some aspects it does not add new knowledge on the study of theileriosis, except for some aspects such as feeding conditions of the animals. The collected data are correctly and clearly presented in the tables. A minimal revision of English is required.

Response. The methodology portion was corrected and information provided accordingly. Similarly, missing information and English corrections were also made.

Attachment Submitted filename: Response Letter.doc Click here for additional data file.

# Peer review: Decision Letter 1

**DOI:** 10.1371/journal.pone.0249417.r003

**Authors:** Shawky M. Aboelhadid

27 Jul 2021

PONE-D-21-07164R1

Epidemiology and molecular characterization of Theileria annulata in cattle from central Khyber Pakhtunkhwa, Pakistan

PLOS ONE

Dear Dr. Jehan Zeb,

Thank you for submitting your manuscript to PLOS ONE. After careful consideration, we feel that it has merit but does not fully meet PLOS ONE’s publication criteria as it currently stands. Therefore, we invite you to submit a revised version of the manuscript that addresses the points raised during the review process.

==============================

ACADEMIC EDITOR: Please try to respond to the suggested comments of the reviewer. 

==============================

Please submit your revised manuscript by Sep 10 2021 11:59PM. If you will need more time than this to complete your revisions, please reply to this message or contact the journal office at plosone@plos.org. When you're ready to submit your revision, log on to https://www.editorialmanager.com/pone/ and select the 'Submissions Needing Revision' folder to locate your manuscript file.

Please include the following items when submitting your revised manuscript:

- A rebuttal letter that responds to each point raised by the academic editor and reviewer(s). You should upload this letter as a separate file labeled 'Response to Reviewers'.
- A marked-up copy of your manuscript that highlights changes made to the original version. You should upload this as a separate file labeled 'Revised Manuscript with Track Changes'.
- An unmarked version of your revised paper without tracked changes. You should upload this as a separate file labeled 'Manuscript'.

If you would like to make changes to your financial disclosure, please include your updated statement in your cover letter. Guidelines for resubmitting your figure files are available below the reviewer comments at the end of this letter.

If applicable, we recommend that you deposit your laboratory protocols in protocols.io to enhance the reproducibility of your results. Protocols.io assigns your protocol its own identifier (DOI) so that it can be cited independently in the future. For instructions see: http://journals.plos.org/plosone/s/submission-guidelines#loc-laboratory-protocols. Additionally, PLOS ONE offers an option for publishing peer-reviewed Lab Protocol articles, which describe protocols hosted on protocols.io. Read more information on sharing protocols at https://plos.org/protocols?utm_medium=editorial-email&utm_source=authorletters&utm_campaign=protocols.

We look forward to receiving your revised manuscript.

Kind regards,

Shawky M. Aboelhadid, PhD

Academic Editor

PLOS ONE

Journal Requirements:

Please review your reference list to ensure that it is complete and correct. If you have cited papers that have been retracted, please include the rationale for doing so in the manuscript text, or remove these references and replace them with relevant current references. Any changes to the reference list should be mentioned in the rebuttal letter that accompanies your revised manuscript. If you need to cite a retracted article, indicate the article’s retracted status in the References list and also include a citation and full reference for the retraction notice.

Additional Editor Comments (if provided):

[Note: HTML markup is below. Please do not edit.]

Reviewers' comments:

Reviewer's Responses to Questions

**Comments to the Author**

1. If the authors have adequately addressed your comments raised in a previous round of review and you feel that this manuscript is now acceptable for publication, you may indicate that here to bypass the “Comments to the Author” section, enter your conflict of interest statement in the “Confidential to Editor” section, and submit your "Accept" recommendation.

Reviewer #1: (No Response)

Reviewer #2: All comments have been addressed

**********

2. Is the manuscript technically sound, and do the data support the conclusions?

The manuscript must describe a technically sound piece of scientific research with data that supports the conclusions. Experiments must have been conducted rigorously, with appropriate controls, replication, and sample sizes. The conclusions must be drawn appropriately based on the data presented. 

Reviewer #1: Yes

Reviewer #2: Yes

**********

3. Has the statistical analysis been performed appropriately and rigorously? 

Reviewer #1: Yes

Reviewer #2: Yes

**********

4. Have the authors made all data underlying the findings in their manuscript fully available?

The PLOS Data policy requires authors to make all data underlying the findings described in their manuscript fully available without restriction, with rare exception (please refer to the Data Availability Statement in the manuscript PDF file). The data should be provided as part of the manuscript or its supporting information, or deposited to a public repository. For example, in addition to summary statistics, the data points behind means, medians and variance measures should be available. If there are restrictions on publicly sharing data—e.g. participant privacy or use of data from a third party—those must be specified.

Reviewer #1: Yes

Reviewer #2: Yes

**********

5. Is the manuscript presented in an intelligible fashion and written in standard English?

PLOS ONE does not copyedit accepted manuscripts, so the language in submitted articles must be clear, correct, and unambiguous. Any typographical or grammatical errors should be corrected at revision, so please note any specific errors here.

Reviewer #1: Yes

Reviewer #2: Yes

**********

6. Review Comments to the Author

Please use the space provided to explain your answers to the questions above. You may also include additional comments for the author, including concerns about dual publication, research ethics, or publication ethics. (Please upload your review as an attachment if it exceeds 20,000 characters)

Reviewer #1: The revised manuscript has been significantly improved, the majority of the reviewers recommendations were addressed. For this reason, I recommend to accept the present manuscript for publication in PlosOne. In order to optimise the quality of the final version of the manuscript, I will however strongly recommend to the authors, the include the following points:

- To mention the tropical theileriosis season(s) in the study region, since this information will position the sampling period (April to September) in regard to the disease season, this information could be relevant to discuss better the sensitivity of blood smears and PCR detection in regard to T. annulata transmission dynamics

- A mention if available in the region or in similar other Pakistani regions on the vector Hyalomma anatolicum ticks dynamics

- To add to the discussion possible reasons explaining the higher prevalence recorded in younger animals

In addition, the authors should take care to make the following corrections:

- Line 61: use the valid name of H. scupense instead of H. detritum .

- Line 77: the phrase “This diagnostic procedure has limited use as it cannot detect T. annulata in infected carrier animals” is not coherent with the fact that the animals detected in the present study are presumably carriers since the authors did not mention any clinical cases of tropical theileriosis in the surveyed farms. I will recommend to change the phrase as follow: this diagnostic procedure is not enough sensitive to allow a reliable detection of carrier animals.

- Line 225: the last phrase is redundant since the same information has been mentioned at lines 222-223.

Reviewer #2: The authors followed all indications suggested in the first round of review. All suggestions were properly followed and all requests accommodated.

**********

7. PLOS authors have the option to publish the peer review history of their article (what does this mean?). If published, this will include your full peer review and any attached files.

If you choose “no”, your identity will remain anonymous but your review may still be made public.

**Do you want your identity to be public for this peer review?** For information about this choice, including consent withdrawal, please see our Privacy Policy.

Reviewer #1: No

Reviewer #2: No

[NOTE: If reviewer comments were submitted as an attachment file, they will be attached to this email and accessible via the submission site. Please log into your account, locate the manuscript record, and check for the action link "View Attachments". If this link does not appear, there are no attachment files.]

While revising your submission, please upload your figure files to the Preflight Analysis and Conversion Engine (PACE) digital diagnostic tool, https://pacev2.apexcovantage.com/. PACE helps ensure that figures meet PLOS requirements. To use PACE, you must first register as a user. Registration is free. Then, login and navigate to the UPLOAD tab, where you will find detailed instructions on how to use the tool. If you encounter any issues or have any questions when using PACE, please email PLOS at figures@plos.org. Please note that Supporting Information files do not need this step.

# Author comment: Author response to Decision Letter 1

**DOI:** 10.1371/journal.pone.0249417.r004

1 Aug 2021

R/Dear Academic Editor Dr. Shawky M. Aboelhadid,

Thank you for your letter and the opportunity to revise and submit our manuscript again on ‘EPIDEMIOLOGY AND MOLECULAR CHARACTERIZATION OF THEILERIA ANNULATA IN CATTLE FROM CENTRAL KHYBER PAKHTUNKHWA, PAKISTAN’ The suggestions offered by the reviewer have been immensely helpful and constructive. We are thankful to you, to the reviewers and PLOS ONE scientific platform which support and encourage young researchers’ publications. 

We have addressed all the comments, revised and proofread the manuscript together with reference list to exclude any typos if present. Responses to the journal requirements and reviewer’s comments are included immediately after this letter. The revised manuscript is attached to this email message (track changed and clean copies).

Sincerely,

Dr. Jehan Zeb 

Department of Zoology

Abdul Wali Khan University Mardan, Pakistan

Response to the Journal Requirements:

1. Please review your reference list to ensure that it is complete and correct. If you have cited papers that have been retracted, please include the rationale for doing so in the manuscript text, or remove these references and replace them with relevant current references. Any changes to the reference list should be mentioned in the rebuttal letter that accompanies your revised manuscript. If you need to cite a retracted article, indicate the article’s retracted status in the References list and also include a citation and full reference for the retraction notice.

Response: The whole reference list is carefully reviewed. No retracted reference was found in the list. The references are arranged according to the standard PLOS ONE style template. The reference section is complete and correct.

Response to the Reviewer’s Comments

Reviewer #1: The revised manuscript has been significantly improved, the majority of the reviewers’ recommendations were addressed. For this reason, I recommend to accept the present manuscript for publication in PlosOne. In order to optimise the quality of the final version of the manuscript, I will however strongly recommend to the authors, to nclude the following points:

1. To mention the tropical theileriosis season(s) in the study region, since this information will position the sampling period (April to September) in regard to the disease season, this information could be relevant to discuss better the sensitivity of blood smears and PCR detection in regard to T. annulata transmission dynamics.

Response: The relevant information are addressed in the revised manuscript. The literature about the disease season (s) is inserted in the introduction section and our findings are also explained in the results sections.

2. A mention if available in the region or in similar other Pakistani regions on the vector Hyalomma anatolicum ticks dynamics.

Response: There is no relevant data available about the vector tick H. anatolicum dynamics. All such information will be investigated and addressed soon in our incoming articles.

3. To add to the discussion possible reasons explaining the higher prevalence recorded in younger animals.

Response: We have provided relevant and possible reasons to justify the higher prevalence recorded in younger animals.

In addition, the authors should take care to make the following corrections:

- Line 61: use the valid name of H. scupense instead of H. detritum.

Response: The mentioned species name (H. detritum) was replaced with the valid species name H. scupense. The synonym is removed.

- Line 77: the phrase “This diagnostic procedure has limited use as it cannot detect T. annulata in infected carrier animals” is not coherent with the fact that the animals detected in the present study are presumably carriers since the authors did not mention any clinical cases of tropical theileriosis in the surveyed farms. I will recommend to change the phrase as follow: this diagnostic procedure is not enough sensitive to allow a reliable detection of carrier animals.

Response: The mentioned sentence is rephrased as suggested by reviewer “this diagnostic procedure is not enough sensitive to allow a reliable detection of TT in carrier animals”.

- Line 225: the last phrase is redundant since the same information has been mentioned at lines 222-223.

Response: This portion is reshaped by removing the redundant phrase.

We are thankful to reviewer # 1 for his expert and constructive comments which have improved the quality of our manuscript. 

Reviewer #2: The authors followed all indications suggested in the first round of review. All suggestions were properly followed and all requests accommodated.

Response: Thank you sir for providing us the opportunity to revise and improve our manuscript.

Attachment Submitted filename: Response to Reviewers.doc Click here for additional data file.

# Peer review: Decision Letter 2

**DOI:** 10.1371/journal.pone.0249417.r005

**Authors:** Shawky M. Aboelhadid

31 Aug 2021

Epidemiology and molecular characterization of Theileria annulata in cattle from central Khyber Pakhtunkhwa, Pakistan

PONE-D-21-07164R2

Dear Dr. Jehan Zeb,

We’re pleased to inform you that your manuscript has been judged scientifically suitable for publication and will be formally accepted for publication once it meets all outstanding technical requirements.

Within one week, you’ll receive an e-mail detailing the required amendments. When these have been addressed, you’ll receive a formal acceptance letter and your manuscript will be scheduled for publication.

An invoice for payment will follow shortly after the formal acceptance. To ensure an efficient process, please log into Editorial Manager at http://www.editorialmanager.com/pone/, click the 'Update My Information' link at the top of the page, and double check that your user information is up-to-date. If you have any billing related questions, please contact our Author Billing department directly at authorbilling@plos.org.

If your institution or institutions have a press office, please notify them about your upcoming paper to help maximize its impact. If they’ll be preparing press materials, please inform our press team as soon as possible -- no later than 48 hours after receiving the formal acceptance. Your manuscript will remain under strict press embargo until 2 pm Eastern Time on the date of publication. For more information, please contact onepress@plos.org.

Kind regards,

Shawky M. Aboelhadid, PhD

Academic Editor

PLOS ONE

Additional Editor Comments (optional):

Reviewers' comments:

Reviewer's Responses to Questions

**Comments to the Author**

1. If the authors have adequately addressed your comments raised in a previous round of review and you feel that this manuscript is now acceptable for publication, you may indicate that here to bypass the “Comments to the Author” section, enter your conflict of interest statement in the “Confidential to Editor” section, and submit your "Accept" recommendation.

Reviewer #1: All comments have been addressed

**********

2. Is the manuscript technically sound, and do the data support the conclusions?

The manuscript must describe a technically sound piece of scientific research with data that supports the conclusions. Experiments must have been conducted rigorously, with appropriate controls, replication, and sample sizes. The conclusions must be drawn appropriately based on the data presented. 

Reviewer #1: Yes

**********

3. Has the statistical analysis been performed appropriately and rigorously? 

Reviewer #1: Yes

**********

4. Have the authors made all data underlying the findings in their manuscript fully available?

The PLOS Data policy requires authors to make all data underlying the findings described in their manuscript fully available without restriction, with rare exception (please refer to the Data Availability Statement in the manuscript PDF file). The data should be provided as part of the manuscript or its supporting information, or deposited to a public repository. For example, in addition to summary statistics, the data points behind means, medians and variance measures should be available. If there are restrictions on publicly sharing data—e.g. participant privacy or use of data from a third party—those must be specified.

Reviewer #1: Yes

**********

5. Is the manuscript presented in an intelligible fashion and written in standard English?

PLOS ONE does not copyedit accepted manuscripts, so the language in submitted articles must be clear, correct, and unambiguous. Any typographical or grammatical errors should be corrected at revision, so please note any specific errors here.

Reviewer #1: Yes

**********

6. Review Comments to the Author

Please use the space provided to explain your answers to the questions above. You may also include additional comments for the author, including concerns about dual publication, research ethics, or publication ethics. (Please upload your review as an attachment if it exceeds 20,000 characters)

Reviewer #1: The authors have correctly addressed all the issues raised by reviewers, accordingly the present manuscript could be accepted, as it is sanding, for publication in PLOS ONE.

**********

7. PLOS authors have the option to publish the peer review history of their article (what does this mean?). If published, this will include your full peer review and any attached files.

If you choose “no”, your identity will remain anonymous but your review may still be made public.

**Do you want your identity to be public for this peer review?** For information about this choice, including consent withdrawal, please see our Privacy Policy.

Reviewer #1: No

# Editor report: Acceptance letter

**DOI:** 10.1371/journal.pone.0249417.r006

**Authors:** Shawky M. Aboelhadid

6 Sep 2021

PONE-D-21-07164R2 

Epidemiology and molecular characterization of *Theileria annulata* in cattle from central Khyber Pakhtunkhwa, Pakistan 

Dear Dr. Zeb:

I'm pleased to inform you that your manuscript has been deemed suitable for publication in PLOS ONE. Congratulations! Your manuscript is now with our production department. 

If your institution or institutions have a press office, please let them know about your upcoming paper now to help maximize its impact. If they'll be preparing press materials, please inform our press team within the next 48 hours. Your manuscript will remain under strict press embargo until 2 pm Eastern Time on the date of publication. For more information please contact onepress@plos.org.

If we can help with anything else, please email us at plosone@plos.org. 

Thank you for submitting your work to PLOS ONE and supporting open access. 

Kind regards, 

PLOS ONE Editorial Office Staff

on behalf of

Professor Shawky M. Aboelhadid 

Academic Editor

PLOS ONE
